# Identifying the nutrition support nurses’ tasks using importance–performance analysis in Korea: a descriptive study

**DOI:** 10.3352/jeehp.2023.20.3

**Published:** 2023-01-18

**Authors:** Jeong Yun Park

**Affiliations:** Department of Clinical Nursing, University of Ulsan, Seoul, Korea; Hallym University, Korea

**Keywords:** Leadership, Nurses, Nutritional status, Referral and consultation, Republic of Korea

## Abstract

**Purpose:**

Nutrition support nurse is a member of a nutrition support team and is a health care professional who takes a significant part in all aspects of nutritional care. This study aims to investigate ways to improve the quality of tasks performed by nutrition support nurses through survey questionnaires in Korea.

**Methods:**

An online survey was conducted between October 12 and November 31, 2018. The questionnaire consists of 36 items categorized into 5 subscales: nutrition-focused support care, education and counseling, consultation and coordination, research and quality improvement, and leadership. The importance–performance analysis method was used to confirm the relationship between the importance and performance of nutrition support nurses’ tasks.

**Results:**

A total of 101 nutrition support nurses participated in this survey. The importance (5.56±0.78) and performance (4.50±1.06) of nutrition support nurses’ tasks showed a significant difference (t=11.27, P<0.001). Education, counseling/consultation, and participation in developing their processes and guidelines were identified as low-performance activities compared with their importance.

**Conclusion:**

To intervene nutrition support effectively, nutrition support nurses should have the qualification or competency through the education program based on their practice. Improved awareness of nutrition support nurses participating in research and quality improvement activity for role development is required.

## Introduction

### Background/rationale

Hospitals have established multidisciplinary intensive nutrition support teams (NSTs) in response to the growing importance and interest in nutritional support for patient treatment [1]. The Ministry of Health and Welfare of Korea introduced health insurance coverage for intensive nutrition therapy in August 2014, which provided an opportunity to expand the NST’s activities. The NST is a multidisciplinary team of doctors, nurses, dietitians, and pharmacists who provide intensive nutrition support services, such as assessing nutritional status, determining nutritional needs, consulting nutrition treatment, and managing intensive nutrition therapy [1,2]. Here reimbursement for NST is granted when hospitals establish an NST, properly train their members, and have at least one member solely responsible for NST [3]. Doctors, nurses, and pharmacists are among the health care professionals who can take on this role. However, assigning doctors to the role increases the budget, andit is challenging to hire pharmacists or dietitians due to labor shortages. Thus, nurses often work as NST personnel [4,5]. After completing a required nutritional therapy educational training course and registering with the Health Insurance Review and Assessment Service, they are recognized for their activities as members of the intensive NST [3,6]. A nurse’s responsibilities include providing nutritional support nursing as an advanced practice nurse, consulting with other health care providers, educating patients, caregivers, and medical staff, participating in research and quality improvement (QI) activities, and performing administrative functions as nutritional support nurses (NSNs) [7,8]. Still, other NST members lack understanding of the nurse’s role and responsibilities. One-day educational training is not enough to acquire the competency for nutrition support nursing. Continuing education or training for NSNs has been emphasized to standardize NSNs’ tasks in NSTs.

As the expected roles of nurses in teams grew [8,9], so did interest in standardizing nursing practices in the NST. Practice guidelines for each NST member have already been published by the *American Society of Parenteral and Enteral Nutrition* or the *European Society of Parenteral and Enteral Nutrition* [7,10]. In Korea, hospitals have an NST manual through health care accreditation, but its contents should be reviewed due to differences from actual tasks performed. However, few studies on nutrition support nursing and the role of NSNs in the NST have been conducted [4,5,10]. Thus, it is necessary to investigate and analyze the tasks of NSNs as NST personnel and examine the performance and importance of tasks among NSNs in Korea.

### Objectives

This study aims to identify the importance and performance of tasks among NSNs in Korea, and to find out the important tasks to manage and suggest focus using the importance-performance analysis.

## Methods

### Ethics statement:

This study was approved by the Institutional Review Board of Univestiy of Ulsan (1040968-A2018-012). Informed consent was obtained in the first section of the online survey from participants.

### Study design

A descriptive study was done using a online survey. Participants opened the link to the online survey posted on the website. The study was described according to the STROBE (Strengthening the Reporting of Observational Studies in Epidemiology) statement (https://www.strobe-statement.org) [11].

### Setting

This study was conducted among nutrition support nurses who are members of the nutrition support teams. In 2018, there were 42 tertiary general hospitlas or 311 general hositals that could claim the nutrition support team consultation fees [4]. Data were collected from October 12 to November 31, 2018, and an online survey via Google Forms (Google LLC) of 101 subjects who responded to the electronic survey was finally analyzed.

### Participants

The subjects of this study were 101 NST nurses in South Korea, who were informed about the study’s purpose and agreed to participate. The number of target population could not be accuragely determined, assuming that at least one NST nurse is assigned to each hospital, the total number of NST nurses might be estimated 353. Some hospitals have separate pediatric NSTs, so the number may be larger. There was no exclusion criterion.

### Variables

A total of 36 items of the questionnaire were divided into 5 subscales: nutrition-focused support care, education and counseling, consultation and coordination, research and QI, and leadership. Those 5 subscales were variables.

### Data sources/measurement

The questionnaire was developed based on the NST tasks from the hospital manual or nutrition support nurses’ standard practice [7,12,13]. NSNs’ tasks were divided into 5 subscales: nutrition-focused support care (7 items), education and counseling (6 items), consultation and coordination (7 items), research and QI (4 items), and leadership (12 items).

A total of 36 items were examined for content validity by 1 professor in the nursing department, 1 doctor as a director in NST, and 3 nurses with more than 10 years of clinical experience and 5 years of experience in NST. The content validity index (CVI) was used to determine content validity. If the ratio was more than 0.8, the item was deemed to have high content validity. Each questionnaire’s CVI was mor than 0.8, and the average CVI was 0.91. Participants evaluated their importance and performance using a self-reported questionnaire with a 7-point Likert scale. The measurement of task importance ranges from 1 (not at all) to 7 (strongly agreed), and the measurement of task performance ranges from 1 (not at all) to 7 (strongly agreed). The greater the number, the greater the importance of the items and the higher the performance (Supplements 1, 2). The reliability of the measurements used in this study showed a Cronbach’s α of 0.97 for importance and 0.95 for performance of the NSNs’ tasks. Ras response data from participants are available at Dataset 1.

### Bias

There may be selection bias. The study included only participants who accessed an online survey.

### Study size

A priori sample size calculation was not possible because we had no reliable estimate for importance and performance of nutrition support nurses’ tasks. Therefore, we performed a post hos power estimation of our data. The averages and standards deviation of importance and performance of nutrition support nurses’ tasks were 5.56±0.78 and 4.50±1.06, respectively. And, the correlation coefficient between importance and performance of nutrition support nurses’ tasks was 0.48. With an α of 0.05 and participants number of 101, post hoc power analysis indicated our study had over 99% power. The post hoc power analysis was performed with use of G*Power software (ver. 3.1.9.7; Heinrich-Heine-Universität Düsseldorf).

### Statistical methods

The collected data were analyzed using the IBM SPSS ver. 23.0 program (IBM Corp.). The detailed data analysis methods were as follows. The general characteristics of the study subjects and their workplaces were analyzed using numbers, percentages, means, and standard deviation. Furthermore, the importance and performance of the NSNs’ tasks were analyzed using means and standard deviation. The paired t-test was used to examine the differences in the importance and performance of the NSNs’ tasks. Finally, the importance and performance of the NSNs’ tasks were evaluated using the importance–performance analysis method. The importance–performance analysis method can quickly identify research results by measuring the importance and performance of the assessment factors, schematizing the analysis results in quadrants, and giving meaning based on their location. Of the 4 quadrants, quadrant 1 refers to “doing great,” as it has high importance and performance. Quadrant 2 is a “focus here” zone with high importance but low-performance. Quadrant 3 is a “low priority” area with low importance and performance. Finally, quadrant 4 has a high level of performance despite its low importance and refers to “overdone” [14].

## Results

### Demographic and workplace characteristics

The demographic and workplace characteristics of the subjects are shown in Table 1. Of the total 101 nurses (98.0% women), 50.5% were in their 30s, 29.7% were in their 40s, and 9.9% were in their 20s and 50s. Forty-four nurses (43.6%) had a bachelor’s degree. Fifty-four nurses (53.5%) were part-time at NST. The mean total clinical experience was 15.9±7.5 and 2.5±2.4 years for a career in NST. The average score of the perceived effect of nutrition education on practice was 3.85±0.97 out of 5. Forty-nine nurses (48.5%) worked in a tertiary hospital. The workplace of 82 nurses (81.2%) was located in urban areas. Forty-seven nurses (46.5%) had a separate NST office.

### Difference between the importance and performance of NSNs’ tasks

As shown in Table 2, the it revealed that the importance score of all categories was significantly higher than the performance score (P<0.001). The average importance and performance scores for NSNs’ tasks were 5.56±0.78 and 4.50±1.06, respectively.

The average score of importance for nutrition-focused support care, education and counseling, consultation and coordination, research and QI, and leadership, a subcategory of tasks, were 6.00±0.83, 5.52±0.94, 5.52±0.94, 5.36±1.06, and 5.42±0.82, respectively. The average score of performance for nutrition-focused support care, education and counseling, consultation and coordination, research and QI, and leadership, a subcategory of tasks, were 5.43±1.25, 3.64±1.23, 4.84±1.34, 3.85±1.38, and 4.40±1.18, respectively.

The average score of importance of each item was highest in “participation in a nutrition care plan” (6.32±0.97) and “reply to the formal NST consultation” (6.32±1.02). “Accounting of NST” demonstrated lowest importance with 4.67±1.39. The average score of performance of each item was highest in “reply the formal NST consultation” (5.99±1.65). “Presentation of the research results” demonstrated lowest performance with 2.83±1.63.

### Importance–performance analysis of NSNs’ tasks

**Fig. 1.**
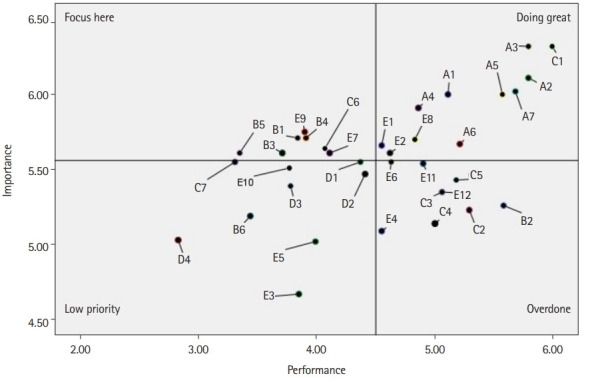
Importance–performance analysis: importance and performance of task items. (1) Doing great: A1, patient assessment: individual patients round; A2, patient assessment: electronic medical record (EMR) review; A3, participation in a nutrition care plan; A4, evaluation of the nutrition care: individual patients round; A5, evaluation of the nutrition care: EMR review; A6, get ready the patient list for NST round; A7, attend the NST round. (2) Focus here: B1, development of education leaflet; B3, education for patient/guardian; B4, education for nurses; B5, education for physician or non-medical persons; C6, ask medical staff or non-medical staff for the consult; E7, participation in process development; E9, participation in guideline development and revision; C1, reply to the formal NST consultation; E1, evaluation of equipment used in the enteral nutrition care process; E2, evaluation of equipment used in the parenteral nutrition care process; E8, environment management: computer system or program. (3) Low priority: B6, educator at symposium or professional organization; C7, review feeding formulations and equipment; D1, research plan, literature review, and data collection; D2, statistics and report; D3, research and quality improvement activities; D4, presentation of the research results; E3, accounting of NST; E5, participation of conference/symposium support work; E10, development of checklist or evaluation form. (4) Overdone: B2, preparation for education; C2, announce NST round and identify the attendees; C3, preparation of NST meeting; C4, announce the conference and identify the attendees; C5, participation of NST meeting; E4, get written various documentations; E6, participation in policy development meeting; E11, participation in conference as presenter or audience; E12, professional organization activities as president or committee.

Importance–performance analysis of NSNs’ tasks is shown in Fig. 1. On average, the importance–performance matrix was divided into 4 quadrants, each with 5.56 importance points and 4.50 performance points. Seven items including “reply to the formal NST consultation” (C1) were shown in quadrant 1. Eleven items including “development of education leaflet” (B1) were located in quadrant 2. Nine items including “presentation of the research results” (D4) were located in quadrant 3. Lastly, nine items including “announce NST round and identify the attendees” (C2) are located in quadrant 4.

## Discussion

### Key results

This study aimed to identify the importance and performance of tasks among NSNs in Korea, and to find out the the important tasks to manage and suggest focus using the importance-performance analysis. The study showed there was a significant difference in all items between the importance and performance of NSNs’ tasks, with 5.56±0.78 and 4.50±1.06, respectively. An importance–performance analysis suggested NSNs perceived research and QI activities to be relatively insignificant and rarely performed and the most important NSNs’ tasks to focus on are “participation in nutrition care plan” and “reply the formal NST consultation”.

### Interpretation

This study was conducted better to understand the importance and performance of NSNs’ tasks. This result indicated that NSNs perceived the importance more highly than the performance. It is necessary to analyze this result in light of work intensity or nurse staffing, which can cause differences in importance and performance. Moreover, it is required to develop strategies for reducing unnecessary tasks while increasing the performance of essential tasks.

An importance–performance analysis was used in this study to identify management strategies for the NSNs’ tasks. As a result, 11 items were confirmed to be the tasks that they perceived as important and could perform well at the same time, including “patient assessment: electronic medical record (EMR) review,” “participation in a nutrition care plan,” “evaluation of the nutrition care: EMR review,” “attending the NST round,” and “replying to the formal NST consultation.” Here, the NST’s activities are included as evaluation criteria in hospital health care accreditation, emphasizing the role and practice of NST members. In this regard, NSNs in NSTs appear to have perceived the importance of their tasks highly and exhibit high levels of performance accordingly. Additionally, nurses must complete a minimally-required nutrition education course to be approved for reimbursement for therapy NST [3,4], which includes the NST’s roles and the roles and tasks of each NST member. The participation of nurses in this education will provide them with an opportunity to recognize their roles and tasks as well as their performance. Unfortunately, compulsory education is only provided once, with no further education required. Therefore, advanced continuing education courses for NSNs are required.

Education, counseling/consultation, and participation in developing their processes and guidelines were identified as low-performance activities compared with their importance. Because education and counseling are independent and specialized tasks of NSNs that directly impact intensive nutrition support services, supportive measures are required to educate NSNs on the importance of their tasks and assist them in making time for education and counseling in the workplace. Moreover, because consultation activities are an important task for collaborations within a multidisciplinary team, it is necessary to provide specialized education to identify and improve the factors that disrupt the performance of consultation tasks.

Activities, such as preparing for education, announcing NST rounds and identifying their attendees, preparing NST meetings, announcing conferences and identifying their attendees, or completing various documents, showed higher levels of performance than their importance. This finding implies that NSNs performed liaison or trivial administrative tasks as high-frequency performance items by acting as a mediator in a multidisciplinary team. Tertiary hospitals, for example, allow NSNs to focus on their original roles by hiring administrative staff to assist with these tasks. Therefore, the placement of these administrative support personnel must be considered.

With the growing importance of nutritional support services in a clinical setting, the NST’s roles have greatly expanded. NSNs must have essential competencies to perform tasks, such as nutrition support nursing, consult, cooperation and coordination, research, and QI activities. Therefore, structured curriculums are urgently needed to develop these competencies. Moreover, as a motivational strategy, it is necessary to emphasize the importance of nutrition support nursing in clinical settings and provide information that allows NSNs to understand the nature of patients and their contribution.

### Comparison with previous studies

In this study, NSNs perceived research and QI activities to be relatively insignificant and rarely performed. It is consistent with the findings of a study that examined the frequency of task performance among South Korean professional medical support staff, in which the staff showed the lowest performance [15]. Low importance and performance areas denote low priority areas but should be gradually improved. Despite the high importance of studies and QI activities in ensuring patient safety and treatment quality [9,12], NSNs did not appear to recognize this importance. Emphasizing the importance of this task should come first. Additionally, participation in research is required to demonstrate the performance of nutritional support services, and hospitals should seek measures to improve NSN research competency.

### Limitations and suggestions

This study targeted only nutrition support nurses who accessed an online survey, there is a limitation in generalizing these results to all nutrition support nurses. It only identified tasks based on perception by NSNs. In a further study, as a member of NST, a multidisciplinary team, it is necessary to consider the views of other members of the work performed by NSN.

### Generalizability

The study participants were working at various hospital type and region, which suggests that it reflects tasks of NSNs. The results of this study may be applied to NSNs in Korea.

### Implications

The following task management strategies are proposed based on the above results. A system should be provided to assist NSNs in recognizing and performing the importance of education, counseling, and consultation, which were identified as areas for intensive improvement among NSNs’ tasks. It is also necessary to reduce NSNs’ repetitive and simple administrative tasks and find ways to provide administrative staff to improve their tasks. This study is significant in that it provides basic data for strategy planning by performing a comparative analysis of the importance and performance of NSNs’ tasks.

### Conclusion

Nutrition support nurse is a member of a nutrition support team and plays a role in nutritional care. For nutrition support nurses to intervene nutrition support effectively, they should have the qualification or competency through the education program based on their practice. It is essential for NSNs to reduce unnecessary administrative management tasks to implement effective nutrition support services. Improved awareness of NSNs participating in research and QI activity for role development is required.

## Figures and Tables

**Table 1.** Demographic characteristics and workplace characteristics of participants (n=101)

Characteristic	Category	Value
Gender	Female	99 (98.0)
	Male	2 (2.0)
Age (yr)		39.0±7.2
	<30	10 (9.9)
	30–39	51 (50.5)
	40–49	30 (29.7)
	≥50	10 (9.9)
Education	Associate	11 (10.9)
	Bachelor	44 (43.6)
	Master degree	35 (34.7)
	Doctoral degree	11 (10.9)
Department of hospital	NST	27 (26.7)
	Nursing department	64 (63.4)
	Medical or dietitian department	10 (9.9)
Employment type in NST	Full-time	47 (46.5)
	Part-time	54 (53.5)
Clinical experience (yr)		15.9±7.5
	<5	3 (3.0)
	5–9	21 (20.8)
	10–14	23 (22.8)
	15–19	27 (26.7)
	≥20	27 (26.7)
Career in NST (yr)		2.5±2.4
	<1	24 (23.8)
	1–3	54 (53.5)
	≥4	23 (22.8)
Participation in education related to nutrition^a^	Certified multidisciplinary program	90 (99.1)
	Continue education program for nurses	27 (26.7)
	Academic program	8 (7.9)
Perceived effect of education for practice (1–5)		3.85±0.97
Type of hospital	Tertiary	49 (48.5)
	General: >300 beds	41 (40.6)
	General: 100–300 beds	11 (10.9)
Location of hospital	Urban	82 (81.2)
	Rural	19 (8.8)
NST consultation fee	Yes	80 (79.2)
	No	21 (20.8)
Existence of manual for nutrition care	Yes	51 (50.5)
	No	50 (49.5)
Separated NST office	Yes	47 (46.5)
	No	54 (53.5)

Values are presented as number (%) or mean±standard deviation. NST, nutrition support team.

a) Multiple responses.

**Table 2.** Importance and performance of nutrition support nurses’ tasks (n=101)

Task	Importance	Performance	t-value	P-value
Overall	5.56±0.78	4.50±1.06	11.27	<0.001
Nutrition-focused support care				
A1. Patient assessment: individual patients round	6.00±1.09	5.11±1.90	5.47	<0.001
A2. Patient assessment: EMR review	6.11±1.09	5.79±1.59	2.55	0.012
A3. Participation in a nutrition care plan	6.32±0.97	5.79±1.29	3.76	<0.001
A4. Evaluation of the nutrition care: individual patients round	5.91±1.76	4.86±1.78	6.53	<0.001
A5. Evaluation of the nutrition care: EMR review	6.00±1.10	5.57±1.64	3.01	0.003
A6. Get ready the patient list for NST round	5.67±1.41	5.21±1.97	2.59	0.011
A7. Attend the NST round	6.02±1.02	5.68±1.65	2.17	0.032
Subtotal	6.00±0.83	5.43±1.25	5.40	<0.001
Education and counseling				
B1. Development of education leaflet	5.71±1.04	3.84±1.57	10.87	<0.001
B2. Preparation for education	5.26±1.11	5.58±1.61	10.39	<0.001
B3. Education for patient/guardian	5.61±1.00	3.71±1.58	12.26	<0.001
B4. Education for nurses	5.71±1.04	3.91±1.64	10.85	<0.001
B5. Education for physician or non-medical persons	5.61±1.05	3.35±1.47	14.65	<0.001
B6. Educator at symposium or professional organization	5.19±1.30	3.44±1.76	10.40	<0.001
Subtotal	5.52±0.94	3.64±1.23	14.76	<0.001
Consultation and coordination				
C1. Reply to the formal NST consultation	6.32±1.02	5.99±1.65	2.22	0.028
C2. Announce NST round and identify the attendees	5.23±1.35	5.29±1.95	−2.96	0.768
C3. Preparation of NST meeting	5.35±1.26	5.06±1.85	1.54	0.128
C4. Announce the conference and identify the attendees	5.14±1.22	5.00±1.93	0.74	0.462
C5. Participation of NST meeting	5.43±1.15	5.18±1.74	1.51	0.134
C6. Ask medical staff or non-medical staff for the consult	5.64±1.02	4.07±1.61	9.72	<0.001
C7. Review feeding formulations and equipment	5.55±1.04	3.31±1.85	13.48	<0.001
Subtotal	5.52±0.94	4.84±1.34	5.74	<0.001
Research and quality improvement				
D1. Research plan, literature review, and data collection	5.55±1.12	4.37±1.67	7.53	<0.001
D2. Statistics and report	5.47±1.10	4.41±2.01	5.14	<0.001
D3. Research and quality improvement activities	5.39±1.22	3.78±1.77	9.18	<0.001
D4. Presentation of the research results	5.03±1.32	2.83±1.63	12.71	<0.001
Subtotal	5.36±1.06	3.85±1.38	10.51	<0.001
Leadership				
E1. Evaluation of equipment used in the enteral nutrition care process	5.66±1.07	4.55±1.60	7.92	<0.001
E2. Evaluation of equipment used in the parenteral nutrition care process	5.61±1.10	4.62±1.57	6.91	<0.001
E3. Accounting of NST	4.67±1.39	3.85±2.28	4.03	<0.001
E4. Get written various documentation	5.09±1.34	4.55±2.19	2.67	0.009
E5. Participation of conference/symposium support work	5.02±1.27	3.99±2.00	5.36	<0.001
E6. Participation in of policy development meeting	5.55±1.04	4.63±1.70	5.66	<0.001
E7. Participation in process development	5.61±1.05	4.11±1.79	9.05	<0.001
E8. Environment management: computer system or program	5.70±1.09	4.83±1.88	5.26	<0.001
E9. Participation in guideline development and revision	5.75±1.09	3.90±1.80	9.18	<0.001
E10. Development of checklist or evaluation form	5.51±1.21	3.77±1.71	8.87	<0.001
E11. Participation in conference as presenter or audience	5.54±1.15	4.90±1.60	4.47	<0.001
E12. Professional organization activities as president or committee	5.35±1.09	5.06±1.66	1.89	0.061
Subtotal	5.42±0.82	4.40±1.18	9.54	<0.001

Values are presented as mean±standard deviation, unless otherwise stated. EMR, electronic medical record; NST, nutrition support team.

## Footnotes

**Data availability**
Data files are available from Harvard Dataverse: https://doi.org/10.7910/DVN/SXPK1W Dataset 1. Raw response data from participants on the survey questionnaires used for identifying the nutrition support nurses' tasks using importance-performance analysis in Korea.

## Supplementary materials

Supplementary files are available from Harvard Dataverse: https://doi.org/10.7910/DVN/SXPK1W

Supplement 1. Survey questionnaires used for identifying the nutrition support nurses’ tasks using importance-performance analysis in Korea in Korean.

Supplement 2. Survey questionnaires used for identifying the nutrition support nurses’ tasks using importance-performance analysis in Korea in English, which are translated from the Korean version and were not used in the survey.

Supplement 3. Audio recording of the abstract.
