# Supplementary material for: Identifying the nutrition support nurses’ tasks using importance–performance analysis in Korea: a descriptive study
Source: J Educ Eval Health Prof. 2023 Jan 18;20:3. doi: 10.3352/jeehp.2023.20.3 (PMC9935079; doi:10.3352/jeehp.2023.20.3)
Supplement: Supplementary file 2 — Supplement 1. Survey questionnaires used for identifying the nutrition support nurses’ tasks using importance-performance analysis in Korea in Korean. [file jeehp-20-03-suppl1.pdf]

안녕하십니까?

본 설문지는 영양집중지원팀 간호사의 업무 내용을 조사하고, 수행하고 있는 업무의 빈도, 중요도, 난이도를 자세히 알아보기 위해 작성되었습니다. 본 설문지를 통해 영양집중지원팀 간호사의 업무를 이해하고, 간호사의 역할 확립과 업무 수행을 위한 표준화된 교육 프로그램 개발을 돕는 것입니다. 귀하의 답변은 총계로 사용되며 개별 응답으로 평가되지 않습니다. 따라서 가능하면 솔직하게 답변해 주십시오. 정답이나 오답은 없습니다.
본 연구는 자발적으로 참여하는 것으로 참여하지 않더라도 귀하에게 어떠한 불이익도 없을 것입니다. 귀하께서 작성하실 설문지의 작성에 소요되는 예상시간은 10-15분입니다. 귀하께서 연구 참여를 원하지 않으실 경우에는 언제든지 연락해주시면 즉시 중단을 하도록 하겠습니다.
항상 행복하시고 건강하시길 기원합니다.
감사합니다.
다시 한번 귀하의 협조에 감사드립니다.

1. 업무의 빈도

귀하가 수행하는 업무에 대한 빈도를 알아보고자 합니다. 자주 수행하는 업무일수록 높은 점수이며, 점수의 범위는 1점부터 7점까지입니다. 다음 항목에 대해 귀하의 생각에 일치하는 곳에 답해 주십시오.

항목		전혀 하지 않는다	하지 않는다	하지 않은 편이다	보통이다	하는 편이다	한다	자주 한다
1.	Nutrition support team (NST) 의뢰 환자 자료 수집: 직접 방문	1	2	3	4	5	6	7
2.	NST 의뢰 환자 자료 수집: 의무기록 감사	1	2	3	4	5	6	7
3.	영양지원 계획수립: 담당 의료진, NST 팀원 간 의사소통	1	2	3	4	5	6	7
4.	NST 회신서 작성	1	2	3	4	5	6	7
5.	NST 의뢰 환자 이행 평가: 직접 방문	1	2	3	4	5	6	7
6.	NST 의뢰 환자 이행 평가: 의무기록 감사	1	2	3	4	5	6	7
7.	EN 관련 의료장비 관찰/평가	1	2	3	4	5	6	7
8.	PN 관련 의료장비 관찰/평가	1	2	3	4	5	6	7
9.	환자회진 자료 준비(환자명단 등)	1	2	3	4	5	6	7
10	회진 참여 공지 및 참석자 파악	1	2	3	4	5	6	7
11	환자회진 참여	1	2	3	4	5	6	7
12	부서 회의 자료 준비(회의안건 등)	1	2	3	4	5	6	7
13	부서 회의 공지 및 참석자 파악	1	2	3	4	5	6	7

14	부서 회의 참여(부서 운영계획 수립 포함)	1	2	3	4	5	6	7
15	팀 회계업무	1	2	3	4	5	6	7
16	회의록, 협조전 등 각종 공문서 작성	1	2	3	4	5	6	7
17	행사계획 수립 및 운영(학술대회, 심포지엄 등)	1	2	3	4	5	6	7
18	영양지원 관련 교육자료 개발	1	2	3	4	5	6	7
19	영양지원 관련 교육계획 수립: 요구도 조사, 장소 예약, 대상자 모집, 교육자료 준비	1	2	3	4	5	6	7
20	영양지원 관련 교육 제공: 환자/보호자 대상	1	2	3	4	5	6	7
21	영양지원 관련 교육 제공: 간호사 대상	1	2	3	4	5	6	7
22	영양지원 관련 교육 제공: 타의료진 대상	1	2	3	4	5	6	7
23	원내·외 강사활동(실습, 보수교육, 병동 컨퍼런스, 연수강좌 등)	1	2	3	4	5	6	7
24	자문 및 상담(환자/의료진 포함)	1	2	3	4	5	6	7
25	영양지원정책 관련 회의 참여(원내·외 포함)	1	2	3	4	5	6	7
26	영양지원정책 프로세스 개발 참여	1	2	3	4	5	6	7
27	전산프로그램 개발/수정 요청 및 관리	1	2	3	4	5	6	7
28	영양제제 및 관련 물품 심의	1	2	3	4	5	6	7
29	문헌리뷰, 자료수집	1	2	3	4	5	6	7
30	통계자료 작성 및 보고	1	2	3	4	5	6	7
31	영양실무 표준지침 개발 및 개정	1	2	3	4	5	6	7
32	각종 양식 개발(체크리스트, 평가지 등)	1	2	3	4	5	6	7
33	임상연구/ 질향상 활동 참여(계획~평가)	1	2	3	4	5	6	7
34	결과 발표 및 출판(학술대회, 저널 등)	1	2	3	4	5	6	7
35	학술행사 참여(학술대회, 연수강좌, 심포지엄, 워크숍 등)	1	2	3	4	5	6	7
36	원내·외 위원회 활동(NST위원회, 보험위원회 등)	1	2	3	4	5	6	7

2. 업무의 중요도

귀하가 수행하는 업무에 대한 중요도를 알아보고자 합니다. 중요하다고 생각하는 업무일수록 높은 점수이며, 점수의 범위는 1점부터 7점까지입니다. 다음 항목에 대해 귀하의 생각에 일치하는 곳에 답해 주십시오.

항목	전혀 중요하지 않다	중요하지 않다	중요하지 않은 편이다	보통이다	중요한 편이다	중요하다	매우 중요하다
1. NST 의뢰 환자 자료 수집: 직접 방문	1	2	3	4	5	6	7
2. NST 의뢰 환자 자료 수집: 의무기록 감사	1	2	3	4	5	6	7
3. 영양지원 계획수립: 담당 의료진, NST 팀원 간 의사소통	1	2	3	4	5	6	7
4. NST 회신서 작성	1	2	3	4	5	6	7
5. NST 의뢰 환자 이행 평가: 직접 방문	1	2	3	4	5	6	7
6. NST 의뢰 환자 이행 평가: 의무기록 감사	1	2	3	4	5	6	7
7. EN 관련 의료장비 관찰/평가	1	2	3	4	5	6	7
8. PN 관련 의료장비 관찰/평가	1	2	3	4	5	6	7
9. 환자회진 자료 준비(환자명단 등)	1	2	3	4	5	6	7
10 회진 참여 공지 및 참석자 파악	1	2	3	4	5	6	7
11 환자회진 참여	1	2	3	4	5	6	7
12 부서 회의 자료 준비(회의안건 등)	1	2	3	4	5	6	7
13 부서 회의 공지 및 참석자 파악	1	2	3	4	5	6	7
14 부서 회의 참여(부서 운영계획 수립 포함)	1	2	3	4	5	6	7
15 팀 회계업무	1	2	3	4	5	6	7
16 회의록, 협조전 등 각종 공문서 작성	1	2	3	4	5	6	7
17 행사계획 수립 및 운영(학술대회, 심포지엄 등)	1	2	3	4	5	6	7
18 영양지원 관련 교육자료 개발	1	2	3	4	5	6	7
19 영양지원 관련 교육계획 수립: 요구도 조사, 장소 예약, 대상자 모집, 교육자료 준비	1	2	3	4	5	6	7
20 영양지원 관련 교육 제공: 환자/보호자 대상	1	2	3	4	5	6	7
21 영양지원 관련 교육 제공: 간호사 대상	1	2	3	4	5	6	7
22 영양지원 관련 교육 제공: 타의료진 대상	1	2	3	4	5	6	7
23 원내·외 강사활동(실습, 보수교육, 병동 컨퍼런스, 연수강좌 등)	1	2	3	4	5	6	7

24	자문 및 상담(환자/의료진 포함)	1	2	3	4	5	6	7
25	영양지원정책 관련 회의 참여(원내 · 외 포함)	1	2	3	4	5	6	7
26	영양지원정책 프로세스 개발 참여	1	2	3	4	5	6	7
27	전산프로그램 개발/수정 요청 및 관리	1	2	3	4	5	6	7
28	영양제제 및 관련 물품 심의	1	2	3	4	5	6	7
29	문헌리뷰, 자료수집	1	2	3	4	5	6	7
30	통계자료 작성 및 보고	1	2	3	4	5	6	7
31	영양실무 표준지침 개발 및 개정	1	2	3	4	5	6	7
32	각종 양식 개발(체크리스트, 평가지 등)	1	2	3	4	5	6	7
33	임상연구/질향상 활동 참여(계획-평가)	1	2	3	4	5	6	7
34	결과 발표 및 출판(학술대회, 저널 등)	1	2	3	4	5	6	7
35	학술행사 참여(학술대회, 연수강좌, 심포지엄, 워크숍 등)	1	2	3	4	5	6	7
36	원내 · 외 위원회 활동(NST위원회, 보험위원회 등)	1	2	3	4	5	6	7

▶ 다음은 귀하의 일반적 사항에 대한 질문입니다. 각 질문에 답을 작성하거나 해당 항목을 선택하여 주십시오.

소속기관: _________

나이: 만 _____ 세

성별: ☐ 남 ☐ 여

최종 학력: ☐ 전문대 졸업 ☐ 대학 졸업 ☐ 석사 ☐ 박사

임상 경력: ________년 ______개월

현재 업무(부서) 근무 경력: _______년 ______개월

다른 업무와 NST 간호사를 겸직하고 있습니까? ☐ 예(구체적으로 기술:____________) ☐ 아니오

영양 관련 교육 참석: ☐ 예(구체적으로 기술:___________) ☐ 아니오

영양집중치료료 산정 여부: ☐ 예 ☐ 아니오(이유를 구체적으로 기술:_________________)

NST 전담간호사를 위한 직무기술서(지침서)가 구입되어 있습니까? ☐ 예 ☐ 아니오

NST 업무를 위한 개별장소(사무실)가 마련되어 있습니까? ☐ 예 ☐ 아니오

소속병원 규모 : ☐ 예 ☐ 아니오(이유를 구체적으로 기술:_________________)

소속병원의 지역: ______________

수고하셨습니다
