# Supplementary material for: Identifying the nutrition support nurses’ tasks using importance–performance analysis in Korea: a descriptive study
Source: J Educ Eval Health Prof. 2023 Jan 18;20:3. doi: 10.3352/jeehp.2023.20.3 (PMC9935079; doi:10.3352/jeehp.2023.20.3)
Supplement: Supplementary file 3 — Supplement 2. Survey questionnaires used for identifying the nutrition support nurses’ tasks using importance-performance analysis in Korea in English, which are translated from the Korean version and were not used in the survey. [file jeehp-20-03-suppl2.pdf]

Importance and performance of nutrition support nurses' tasks

Performance of Nutrition support nurses' tasks

Items		Not at all			·········			Strongly agreed
1.	Patient assessment: individual patients round	1	2	3	4	5	6	7
2.	Patient assessment: electronic medical record (EMR) review	1	2	3	4	5	6	7
3.	Participation in a nutrition care plan	1	2	3	4	5	6	7
4.	Reply the formal nutrition support team (NST) consultation	1	2	3	4	5	6	7
5.	Evaluation of the nutrition care: Individual patients round	1	2	3	4	5	6	7
6.	Evaluation of the nutrition care: EMR review	1	2	3	4	5	6	7
7.	Evaluation of equipment used in the enteral nutrition care process	1	2	3	4	5	6	7
8.	Evaluation of equipment used in the parenteral nutrition care process	1	2	3	4	5	6	7
9.	Get ready the patient list for NST round	1	2	3	4	5	6	7
10	Announce NST round and identify the attendees	1	2	3	4	5	6	7
11	Attend the NST round	1	2	3	4	5	6	7
12	Preparation of NST meeting	1	2	3	4	5	6	7
13	Announce conference and identify the attendees	1	2	3	4	5	6	7
14	Participation of NST meeting	1	2	3	4	5	6	7
15	Accounting of NST	1	2	3	4	5	6	7
16	Get written various documentation	1	2	3	4	5	6	7
17	Participation of conference/symposium support work	1	2	3	4	5	6	7
18	Development of education leaflet	1	2	3	4	5	6	7
19	Preparation for education	1	2	3	4	5	6	7
20	Education for patient/guardian	1	2	3	4	5	6	7
21	Education for nurses	1	2	3	4	5	6	7
22	Education for physician or non-medical persons	1	2	3	4	5	6	7
23	Educator at symposium or professional organization	1	2	3	4	5	6	7
24	Ask medical staff or non-medical staff for the consult	1	2	3	4	5	6	7
25	Participation in of policy development meeting	1	2	3	4	5	6	7

26	Participation in process development	1	2	3	4	5	6	7
27	Environment management: computer system or program	1	2	3	4	5	6	7
28	Review feeding formulations and equipment	1	2	3	4	5	6	7
29	Research plan, literature review, and data collection	1	2	3	4	5	6	7
30	Statistics and report	1	2	3	4	5	6	7
31	Participation in guideline development and revision	1	2	3	4	5	6	7
32	Development of checklist or evaluation form	1	2	3	4	5	6	7
33	Research and quality improvement activities	1	2	3	4	5	6	7
34	Presentation of the research results	1	2	3	4	5	6	7
35	Participation of conference as presenter or audience	1	2	3	4	5	6	7
36	Professional organization activities as president or committee	1	2	3	4	5	6	7

Importance of nutrition support nurses' tasks

Items		Not at all ································ Strongly agreed						
1.	Patient assessment: individual patients round	1	2	3	4	5	6	7
2.	Patient assessment: EMR review	1	2	3	4	5	6	7
3.	Participation in a nutrition care plan	1	2	3	4	5	6	7
4.	Reply the formal NST consultation	1	2	3	4	5	6	7
5.	Evaluation of the nutrition care: individual patients round	1	2	3	4	5	6	7
6.	Evaluation of the nutrition care: EMR review	1	2	3	4	5	6	7
7.	Evaluation of equipment used in the enteral nutrition care process	1	2	3	4	5	6	7
8.	Evaluation of equipment used in the parenteral nutrition care process	1	2	3	4	5	6	7
9.	Get ready the patient list for NST round	1	2	3	4	5	6	7
10	Announce NST round and identify the attendees	1	2	3	4	5	6	7
11	Attend the NST round	1	2	3	4	5	6	7
12	Preparation of NST meeting	1	2	3	4	5	6	7
13	Announce conference and identify the attendees	1	2	3	4	5	6	7
14	Participation of NST meeting	1	2	3	4	5	6	7

15	Accounting of NST	1	2	3	4	5	6	7
16	Get written various documentation	1	2	3	4	5	6	7
17	Participation of conference/symposium support work	1	2	3	4	5	6	7
18	Development of education leaflet	1	2	3	4	5	6	7
19	Preparation for education	1	2	3	4	5	6	7
20	Education for patient/guardian	1	2	3	4	5	6	7
21	Education for nurses	1	2	3	4	5	6	7
22	Education for physician or non-medical persons	1	2	3	4	5	6	7
23	Educator at symposium or professional organization	1	2	3	4	5	6	7
24	Ask medical staff or non-medical staff for the consult	1	2	3	4	5	6	7
25	Participation in of policy development meeting	1	2	3	4	5	6	7
26	Participation in process development	1	2	3	4	5	6	7
27	Environment management: computer system or program	1	2	3	4	5	6	7
28	Review feeding formulations and equipment	1	2	3	4	5	6	7
29	Research plan, literature review, and data collection	1	2	3	4	5	6	7
30	Statistics and report	1	2	3	4	5	6	7
31	Participation in guideline development and revision	1	2	3	4	5	6	7
32	Development of checklist or evaluation form	1	2	3	4	5	6	7
33	Research and quality improvement activities	1	2	3	4	5	6	7
34	Presentation of the research results	1	2	3	4	5	6	7
35	Participation of conference as presenter or audience	1	2	3	4	5	6	7
36	Professional organization activities as president or committee	1	2	3	4	5	6	7

▶ **General characteristics**

Age: _____

Gender: ☐ Male ☐ Female

Education: ☐ Associate ☐ Bachelor ☐ Master degree ☐ Doctoral degree

Clinical experience: _____________months

Career in NST: _____________months

Employment type in NST: ☐ Full-time ☐ Part-time

Participation in education related to nutrition: ☐ Yes ☐ No

NST consultation fee: ☐ Yes ☐ No

Existence of manual for nutrition care: ☐ Yes ☐ No

Separated NST office: ☐ Yes ☐ No

Hospital type: ☐ Tertiary ☐ General >300 beds ☐ General 100-300 beds

Hospital location: ☐ Urban ☐ Rural

Thank you
